# Dysmenorrhea among University Health Science Students, Northern Ethiopia: Impact and Associated Factors

**DOI:** 10.1155/2018/9730328

**Published:** 2018-01-21

**Authors:** Teshager Aklilu Yesuf, Nigist Assefa Eshete, Eskinder Ayalew Sisay

**Affiliations:** Department of Pharmacy, College of Health Sciences, Mekelle University, P.O. Box 1871, Mekelle, Ethiopia

## Abstract

**Background:**

It is estimated that more than half of all women in adolescence age suffer from dysmenorrhea and it often interferes with their daily physical and emotional aspects. It is the leading cause of short-term school absenteeism and is associated with a negative impact on academic and daily activities.

**Objectives:**

To investigate impacts of dysmenorrhea, factors associated with it, and its self-management strategies used by health science students.

**Methods:**

A cross-sectional institution based study was conducted among 246 Mekelle University health science students selected by stratified random sampling technique. Data were collected using self-administered semistructured questionnaire. Data were analyzed using SPSS 16.

**Results:**

The prevalence of dysmenorrhea was 71.8%. Participants who had long menstrual cycle interval, long menses flows, and positive family history and who were alcohol users were more likely to had dysmenorrhea. Participants reported that 28.6% feel depressed, 16.2% are absent from class, and 22.9% had poor personal relationship due to dysmenorrhea and 78.2% of them practiced self-medication.

**Conclusion:**

Dysmenorrhea is common among Mekelle University health science students and it is major problem representing the cause of feeling depressed, poor personal relationship, and class absenteeism. Majority of the study participants used self-medication to treat dysmenorrhea.

## 1. Introduction

The term dysmenorrhea refers to severe painful cramping sensation in the lower abdomen often accompanied by sweating, tachycardia, headache, nausea, vomiting, diarrhea, and tremulousness occurring just before or during the menses. It is a common gynecological problem among adolescent females which is severe enough to affect their functioning [1–3].

Since the pain results from uterine vasoconstriction and contractions mediated by prostaglandins, the most reliable and effective treatment of dysmenorrhea is to inhibit prostaglandin synthesis using nonsteroidal anti-inflammatory drugs (NSAIDs) [4]. If symptoms do not respond to NSAIDs for three menstrual periods, combined oral contraceptive pills for three menstrual cycles can be tried [5, 6].

Several studies have shown that prevalence of dysmenorrhea varies greatly depending on methods of data collection. According to a study done in Jordan, the prevalence of dysmenorrhea was 90.1% [7]. In China, dysmenorrhea occurred on 37% of the menstrual dates on average and was unrelated to irregularity of menstrual cycles [8]. The symptoms of primary dysmenorrhea begin a few hours before menstruation begins and may peak during the heaviest flow during menstruation [9]; the most common symptoms are stomach cramp (78.0%), backache (58.9%), and mood change (56.9%) [10].

Younger age, low body mass index, smoking, early menarche, prolonged or aberrant menstrual flow, premenstrual somatic complaints, pelvic infections, psychological disturbance, genetic influence, and a history of sexual assault influence the prevalence and severity of dysmenorrhea [3, 11]. A study conducted in Jordan indicated that underweight, having a low family income, living in a rural area, and family history of dysmenorrhea were associated with dysmenorrhea [9]. A study conducted in Vietnam showed mean age and age at menarche, educational status, and religion were associated with dysmenorrhea [12]. A study conducted in Turkey showed that dysmenorrhea was significantly higher in coffee consumers, females with menstrual bleeding duration greater than or equal to seven days, and those who had a family history of dysmenorrhea [13]. Prevalence of dysmenorrhea decreased with increase in age [14, 15]. An experimental study conducted in Iran showed that regular physical activity significantly reduced dysmenorrhea [16].

Dysmenorrhea affects the physical, psychological, and social status of female adolescents. According to study conducted in India among female medical students who reported dysmenorrhea, 31.67% and 8.68% were frequently missing college and classes, respectively [17]. A study done in South India showed that ibuprofen was taken by 80.95% of students [2] while a study done in Taiwan and Ghana showed that paracetamol was the most effective strategies in relieving dysmenorrhea [15, 18]. According to Egyptian study, fresh ginger was effective in relieving menstrual pain [19].

The objective of this study was to determine the prevalence of dysmenorrhea and assess its impact and management strategies used among Mekelle University health science students.

## 2. Methods and Participants

### 2.1. Study Design

A cross-sectional study was conducted from April to May 2014 in Mekelle University, College of Health Sciences, Northern Ethiopia. There were a total of 535 health science female students (medicine, pharmacy, public health, nursing, and dentistry). Considering 95% confidence interval, 5% margin of error, 10% contingency for nonresponse rate, and 50% expected prevalence of dysmenorrhea, a sample of 246 students was calculated using single proportion formula. A two-step stratified random sampling technique was used to select study participants: sampling from each department and then sampling from each academic year based on appropriate proportion of the respective field of study.

Pretested self-administered semistructured questionnaire which was prepared in English was used to collect data. Ethical clearance was obtained from the institutional review board of Mekelle University and consent was sought from each participant. Data were coded and entered using SPSS 16 (SPSS Inc., Chicago, USA) for analysis. Frequencies, means, and standard deviations were calculated where appropriate for each of the variables. Binary logistic regression was done to determine factors associated with dysmenorrhea and *p* value less than 0.05 was considered statistically significant.

## 3. Results

### 3.1. Participants' Background Information

Out of a total of 246 participants recruited to the study, 4 were excluded because of failure to complete the questionnaire, resulting in a response rate of 98%. The mean age of the study participants was 20.5 ± 1.16 years. Most participants were freshman accounting 74 (30.6%) (Table 1).

Majority of participants 173 (71.8%) experienced dysmenorrhea. More than half of the students (54.4%) reported having a family history of dysmenorrhea (Table 2).

Multivariate logistic regression analysis showed that field of study, alcohol use, family history of dysmenorrhea, and duration of menses flow as well as menstrual cycle interval were significantly associated with dysmenorrhea (Table 3).

Most participants have lower abdominal pain (67%). The majority of participants who had dysmenorrhea experienced their symptoms at the onset of menstruation flow (36.4%). Dizziness was the commonest associated symptom, reported by 27.5% respondents, and loss of appetite was the second commonest associated symptom reported by 23.9% respondents (Table 4).

Most participants, 225 (94.1%), did not smoke cigarettes. There were 27 (11.2%) participants who use alcohol. Majority of participants, 137 (56.8%), in this study have habit of drinking coffee. Participants reported that 28.6% of them feel depressed, 16.2% were absent from class, and 22.9% had poor personal relationship because of dysmenorrhea (Table 5).

Ibuprofen was used by 48% of participants. The majorities of the study participants use bed rest (33.6%) and drink more water or tea (31.6%) as a homemade remedy for dysmenorrhea (Table 6).

## 4. Discussion

In this study, the prevalence of dysmenorrhea was 71.8%. This is similar to what is previously reported by Ghanaian adolescent girls, 74.4% [18]. But dysmenorrhea was diagnosed based solely on a girls' perception of pain, which is difficult to quantify and could be related to nonmenstrual events.

Previous studies showed that the prevalence of dysmenorrhea decreases with increasing age and it is significantly associated with early age of menarche [3, 10, 11]. But age at first menarche had no association in our study. Length of menstrual cycle (*p* = 0.03) and duration of menses (*p* = 0.033) were found to be associated with dysmenorrhea. In China, increased menstrual flows (median 4 days and *p* < 0.05) have been reported to be associated with an increase in the severity of dysmenorrhea [8].

Positive family history of dysmenorrhea (*p* < 0.01) was significantly associated with dysmenorrhea. A similar result was obtained from India [2]. The fact that family history was shown to be a risk factor for dysmenorrhea may be related to the risk for related conditions such as endometriosis and genetic factors [13]. Alcohol drinking was also associated with dysmenorrhea (*p* = 0.016). In this study, regular exercise was not associated with experience of dysmenorrhea. But an experimental study done in Turkey found that participating in physical activity decreased the length of menstruation pain and volume of bleeding [16]. Menstrual pain may result from increased contraction of uterine muscle which is innervated by the sympathetic nervous system. Stress is supposed to increase the sympathetic activity which may lead to the increase of menstrual pain by enhancing the intensity of uterine contraction. So, due to the fact that exercise reduces stress, the sympathetic activity may be decreased. Physical activity also leads to the release of endorphins which are produced by brain and may enhance the pain threshold [16].

Dysmenorrhea is a cause of recurrent short-term school absenteeism in adolescent girls and a cause of work absenteeism in women of reproductive age. An estimated 10–15% of women experience monthly menstrual pain severe enough to prevent normal daily function at school, work, or home [18]. In this study, 28.6% of participants reported they feel depressed which could affect their concentration in class and 16.2% of them reported missing classes. Although dysmenorrhea is not a life-threatening condition on its own, monthly recurrence of severe symptoms represents a significant morbidity.

Primary dysmenorrhea is attributable to increased endometrial synthesis of prostaglandins during menstruation. About 2–4 days before the onset of menstruation, prostaglandins proceed into the uterine muscle and cause uterine contractions that help in the expulsion of the endometrium. NSAIDs are the best established initial therapy for dysmenorrhea as they have direct analgesic effect through inhibition of prostaglandin synthesis and they also decrease the volume of menstrual flow [2]. The practice of self-medication appears to be widespread in the adolescent population with dysmenorrhea. In this study, 78.2% of participants reported self-medication. This result is higher than findings from Ghana (51.5%) [18]. Only 5.8% of the study participants had consulted a physician in our study.

NSAIDs are highly effective in treating dysmenorrhea when they are taken before the onset of menses and continued through day two [18]. But our finding indicated that 73.7% of participants reported taking these medications when pain starts indicating the need for professional consultation.

## 5. Conclusion

Dysmenorrhea is a major problem representing the leading cause of class absenteeism. Females with long menstrual bleeding duration and long menstrual cycle interval, positive family history of dysmenorrhea, and alcohol drinking habit are more likely to experience dysmenorrhea. It is very important to create awareness about the causes and treatment of dysmenorrhea via the education system and media. Health professional consultation must be promoted to help students who have dysmenorrhea.

## Figures and Tables

**Table 1:** Demographic characteristics of participants.

Variables	Frequency	Percent
Department		
Medicine	95	39.3
Pharmacy	24	9.9
Public health	25	10.3
Nursing	81	33.5
Dentistry	17	7.0
Academic year		
First year	74	30.6
Second year	56	23.1
Third year	70	28.9
Fourth Year	36	14.9
Fifth year	6	2.5
Religion		
Orthodox	179	74.0
Muslim	28	11.6
Protestant	31	12.8
Other	4	1.7
Marital status		
Unmarried	222	92.9
Married	17	7.1
Mothers' educational level		
Unable to read and write	26	11.0
Able to read and write	65	27.4
High school	40	16.9
Diploma and above	106	44.7

**Table 2:** Menstrual characteristics of participants, 2014.

Variable	Frequency	Percent
Menstruation cycle		
21 days or less	37	15.4
23 to 34 days	188	78.3
35 or greater days	15	6.2
Menstruation cycle regularity		
Regular	163	67.4
Irregular	79	32.6
Menstruation bleeding duration		
2-3 days	54	22.4
4-5 days	155	64.3
6-7 days	32	13.3
Experience of dysmenorrhea		
Yes	173	71.8
No	68	28.2
Family history of dysmenorrhea		
Yes	129	54.4
No	108	45.6

**Table 3:** Factors associated with dysmenorrhea, 2014.

Variables	AOR (95.0% CI)	*p* value
Field of study		**0.047** ^*∗*^
Medicine	Reference	
Pharmacy	1.95 (0.479, 7.90)	0.352
Public health	1.86 (0.55, 6.30)	0.321
Nursing	0.52 (0.19, 1.38)	0.188
Dentistry	0.05 (0.01, 0.85)	0.039
Alcohol use		**0.016** ^*∗*^
Yes	0.06 (0.01, 0.59)	
No	Reference	
Menstrual cycle		**0.030** ^*∗*^
Less than or equal to 21 days	5.62 (1.01, 31.37)	0.049
22–34 days	1.36 (0.31, 5.99)	0.685
Greater than or equal to 35 days	Reference	
Duration of menses		**0.033** ^*∗*^
2-3 days	4.20 (1.01, 17.61)	0.050
4-5 days	1.34 (0.36, 5.01)	0.663
6-7 days	Reference	
Family history of dysmenorrhea		**0.001** ^*∗*^
Yes	0.27 (0.13, 0.59)	
No	Reference	
Constant	0.06	0.529

^*∗*^Statistically significant at *p* value < 0.05; AOR: adjusted odds ratio.

**Table 4:** Dysmenorrhea associated symptoms and sites of pain, 2014.

Symptoms	Frequency	Percent
Lower abdominal pain	163	67.4
Back pain	126	52.0
Thigh pain	20	8.3
Headache	94	19.8
Dizziness	131	27.5
Nausea, vomiting, and diarrhea	50	10.5
Decreased concentration	87	18.3
Loss of appetite	114	23.9

**Table 5:** Impacts of menstrual pain, 2014.

Impact of menstrual pain	Frequency	Percent
Poor concentration at class	62	11.0
Absent from class	91	16.2
Poor personal relationship	129	22.9
Feel depressed	161	28.6
Limitation of sleeping	36	6.5
Limit your exercise	83	14.8

**Table 6:** Medications and home remedies used for managing dysmenorrhea, 2014.

Management strategies	Frequency	Percent
Ibuprofen	137	48.0
Paracetamol	34	12
Diclofenac	72	25.3
Oral contraceptive pills	2	0.7
Apply pad	71	16
Bed rest	150	33.6
Drink more water or tea	141	31.6
Massage the site of pain	69	15.5
Drink different fluids such as soft drinks especially coca cola	15	3.4
None	40	14
